# A Multi‐Center Real‐World Study of Clinicopathologic Characteristics and Efficacy of the Malignant Mesothelioma in Chinese Population

**DOI:** 10.1111/1759-7714.15533

**Published:** 2025-02-12

**Authors:** Chenrui Sun, Xue Yang, Lan Chen, Zhixin Bie, Runting Kang, Bin Ai, Junling Ma, Zitong Zheng, Haolan Liu, Juanjuan Liu, Jia Zhong, Jiangyong Yu

**Affiliations:** ^1^ Center of Biotherapy, Beijing Hospital, National Center of Gerontology, Institute of Geriatric Medicine Chinese Academy of Medical Sciences Beijing China; ^2^ Key Laboratory of Carcinogenesis and Translational Research (Ministry of Education/Beijing), Department I of Thoracic Oncology Peking University Cancer Hospital & Institute Beijing China; ^3^ Department of Pathology, Beijing Hospital, National Center of Gerontology; Institute of Geriatric Medicine Chinese Academy of Medical Sciences Beijing China; ^4^ Minimally Invasive Tumor Therapies Center, Beijing Hospital, National Center of Gerontology, Institute of Geriatric Medicine Chinese Academy of Medical Sciences Beijing China; ^5^ Department of Oncology, Beijing Hospital, National Center of Gerontology, Institute of Geriatric Medicine Chinese Academy of Medical Science Beijing China; ^6^ Department of Oncology Binzhou Medical University Hospital Binzhou China; ^7^ State Key Laboratory of Molecular Oncology, CAMS Key Laboratory of Translational Research on Lung Cancer, Department of Medical Oncology, National Cancer Center/National Clinical Research Center for Cancer/Cancer Hospital Chinese Academy of Medical Sciences Peking Union Medical College Beijing China

**Keywords:** clinical characteristic, efficacy, epidemiology, malignant mesothelioma, survival analysis

## Abstract

**Objective:**

Malignant mesothelioma (MM) is a rare malignant tumor. To explore the clinicopathological characteristics and efficacy of Chinese population with MM in the real‐world.

**Methods:**

Two hundred and forty‐eight patients diagnosed with MM between September 2007 and August 2024 from three large medical centers (Beijing Hospital, Peking University Cancer Hospital, and Chinese Academy of Medical Sciences Cancer Hospital) were retrospectively analyzed. Kaplan–Meier and Cox regression were performed. Breast cancer gene 1‐associated protein 1 (*BAP1*) status was evaluated.

**Results:**

Chinese population with MM had a lower diagnostic age, higher proportion of youth and female, more advanced stage and lower expression of characteristic markers. The median progression‐free survival (mPFS) and median overall survival (mOS) were 8.90 and 25.60 months for the first‐line treatment, and 3.28 and 19.50 months for the second‐line. The first‐line immunotherapy provided a relatively higher objective response rate (33.3% vs. 20.5%, *p* = 0.402) and a trend to prolong mPFS (12.10 vs. 9.20 months, *p* = 0.345) and mOS (NA vs. 23.90, *p* = 0.185) compared with chemotherapy. Bevacizumab combined with chemotherapy relatively prolonged mPFS (10.47 vs. 7.93 months, *p* = 0.074) and mOS (31.30 vs. 23.20 months, *p* = 0.673) than chemotherapy alone. Carboplatin relatively improved mPFS than cisplatin (10.87 vs. 8.87 months, *p* = 0.185). Age and histologic type were predictors for PFS, and gender, histologic subtype, and CK5/6 were prognosis factors for OS. Briefly, 17.78% patients existed *BAP1* deletions and correlated with OS benefit.

**Conclusion:**

Chinese population with MM present unique clinicopathologic characteristics and could benefit from the first‐line immunotherapy and bevacizumab combined with chemotherapy. Gender, histologic subtype, and CK5/6 are prognosis factors for OS. *BAP1* deletions correlate with OS benefit.

## Graphical abstract

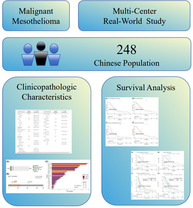

## 1 Introduction

Malignant Mesothelioma (MM) is a rare malignant tumor originating from serosal surfaces, including the pleura, peritoneum, pericardium, testis, and vagina, with more than 80% originating in the pleura [1]. The occurrence of mesothelioma is mainly associated with asbestos exposure. China is the first‐largest asbestos consumer and the second‐largest producer in the world, and there may be more than 1 million Chinese occupationally exposed to asbestos [2]. Although the harmful asbestos has been gradually banned in China, the incidence and mortality rate of MM in China are still increasing due to a 40 years' latency period after asbestos exposure [3]. However, the 2019 Annual Report of China Tumor Registry showed that in 2016, the incidence rate of MM in China was about 0.86/10^6^, which was much lower than the world's average level and might be seriously inconsistent with the actual incidence in China [4]. The difference shows that there is a serious deficiency in the knowledge and diagnosis of MM in China, which leads to a high rate of clinical misdiagnosis and missed diagnosis. It is urgent to well recognize the realistic status of clinical characteristics, efficacy, and survival in the Chinese population with MM.

In the past decade, few studies attempted to investigate the prevalence of MM and evaluate the incidence rate and potential risk factors in a cohort of asbestosis patients in China [2, 5]. The studies demonstrated that Chinese MM had a low relevance with asbestos exposure of 15% [6] and was characterized by a low age at diagnosis, a large proportion of females, and a high prevalence of peritoneal subtype. These results indicated that there might be significant differences in the epidemiology and pathogenic factors of MM in Chinese population.

Presently, the standard systemic treatment of unresectable MM is 4–6 cycles of platinum‐pemetrexed with or without bevacizumab, with an median overall survival (mOS) of 12–16 months [7, 8]. Immune checkpoint inhibitors (ICBs) have also shown efficacy in the treatment of MM. Ipilimumab combined with nivolumab obtained better results compared to conventional chemotherapy, providing a benefit of mOS up to 18.1 months and 26% reduction of death risk, particularly in the non‐epithelioid subtype [9]. Although the real‐world practice and efficacy of the chemotherapy and immunotherapy in regions like France [10], Australia [11], and the whole Europe [12] had been published, the data reflection of patients' characteristics and treatment approaches in the developing country, which has just or even not stopped using asbestos was missing in a larger scale.

Up to now, there have been no publishment of large sample studies on the clinicopathological features and efficacy of Chinese MM patients. In this study, we conducted a retrospectively multicenter real‐world study including Beijing Hospital, Peking University Cancer Hospital, and Chinese Academy of Medical Sciences Cancer Hospital to discuss the epidemiology and clinical outcome of the MM in Chinese Population.

## 2 Patients and Methods

### 2.1 Inclusion Criteria

Chinese population diagnosed with MM by pleural fluid cell pathology, pathological biopsy or surgery at Beijing Hospital, Peking University Cancer Hospital, and Chinese Academy of Medical Sciences Cancer Hospital between September 2007 and August 2024 were included. This study was approved by the medical ethics committee of Beijing Hospital (2022BJYYEC‐211‐02).

Non‐Asian population registered as MM in the Surveillance, Epidemiology, and End Results (SEER) database were retrospectively analyzed as an epidemiological control. Using SEER*stat 8.4.3, patients with (1) diagnosis between 2000 and 2020, (2) ICD‐O3 histologic codes of 9050/3‐9053/3, and exclusions of (1) ethnicity Asian or Pacific Islander, and (2) 38 duplicates were excluded, for a total of 14 328 MM cases. The baseline characteristics including gender, age, disease location, histological type, and clinical stage were recorded.

### 2.2 Variables

Clinical and pathologic data were retrospectively collected from medical records and follow‐up using a predefined template. Fields including (i) demographic characteristics such as gender and age; (ii) Eastern Cooperative Oncology Group performance status (ECOG PS), history of asbestos exposure, smoking, family history of tumors; (iii) details of diagnosis including clinical stage, location of MM, histological type and immunohistochemical (IHC) biomarkers expression; and (v) treatment including detailed therapies and outcomes.

### 2.3 Definitions

Efficacy was determined according to Response Evaluation Criteria in Solid Tumors (RECIST) (version 1.1) [13]. Objective response rate (ORR) was defined as the proportion of patients achieved complete response (CR) or partial response (PR) of all the patients. Disease control rate (DCR) was defined as the proportion of patients with CR, PR, or stable disease (SD).

Progression free survival (PFS): the time from the start of treatment to failure (disease progression or intolerable toxicity) or the last follow‐up visit. OS: the time from the start of treatment or diagnosis until death from any cause or the last follow‐up visit.

### 2.4 Immunohistochemistry (IHC)

IHC staining was performed on 4‐μm thick formalin‐fixed paraffin embedded (FFPE) sections. Antibodies including Calretinin, Cytokeratin 5/6 (CK5/6), Wilms' Tumor gene 1(WT‐1), D2‐40, breast cancer gene 1‐associated protein 1 (BAP‐1), and programmed death‐ligand 1(PD‐L1) were used for IHC staining. Slides were stained on Autostainer Link 48 platform (Agilent Technologies, Santa Clara, CA). Ventana anaplastic lymphoma kinase (ALK) (D5F3) kit was used to test ALK protein expression (Ventana Medical Systems Inc., Tucson, AZ, USA), in accordance with the manufacturer's instructions, which was performed automatically using the Ventana BenchMark XT Stainer (Ventana Medical Systems Inc., Tucson, AZ, USA).

### 2.5 Evaluation of *BAP1* Loss Status

*BAP1* deletion status was evaluated in 45 patients with MM by whole‐exome sequencing (WES) (*n* = 19, 300 × sequencing depth for tumor, 100 × for normal DNA), targeted capture sequencing (*n* = 9), and IHC (*n* = 17). FFPE or fresh tumors or tumor cells isolated from the pleural effusions were collected for tumor DNA extraction. Peripheral blood mononuclear cells (PBMC) were used for germline DNA.

#### 2.5.1 WES By Next Generation Sequencing (NGS)

DNA extraction, library, sequencing, and information analysis were all performed by Beijing Novogene Biotechnology Co. Ltd. Agilent's liquid‐chip capture system was used to efficiently enrich human whole‐exon DNA, and genomic DNA was randomly broken into fragments of 180–280 bp by a Covaris fragmentation machine, and then added to an Illumina for DNA libraries. After passing the quality control, sequencing was performed using the Illumina Novaseq platform PE150 (Pair‐end150bp). Sequenced Reads were stored in FASTQ (fa for short) format and been filtered as follows: reads with adapter deletion; deleting reads containing more than 10% of unrecognized base information; and removal of paired reads when low‐quality (less than 5) bases of single‐ended reads exceeded 50% in length. The clean data were compared to the reference genome (B37) by BWA [14] (Burrows‐Wheeler Aligner). Single‐nucleotide polymorphism (SNP) and InDel sites were identified using SAMtools [15], and SNV and InDel sites were detected using Mutect [16], Strelka [17], and Control‐FREEC [18], respectively.

#### 2.5.2 Targeted‐Capture Sequencing

Targeted cancer mutation profiles were performed using NGS methods including 1021‐gene panel (GenePlus, Beijing, China), 571‐gene panel (AmoyDx, China), or 654‐gene panel (Berry Oncology Co. Ltd). DNA libraries sequencing was carried out utilizing Geneplus Seq‐2000 (GenePlus, Beijing, China) or Illunima Novaseq 6000 system (Illumina Inc., San Diego, California, USA) with paired‐end reads. And sequencing data were base‐called, demultiplexed and filtered and the reads were mapped to the reference human genome (hg19). Mapped reads were deduplicated and the consensus reads were used for somatic variant calling.

### 2.6 Statistical Analysis

Categorical variables were expressed as numbers (%), and the Wilcoxon rank‐sum test or *χ* ^2^ test or Fisher's exact were used to compare the between‐group differences. Survival analysis was performed using the Kaplan–Meier curves and log‐rank test. Factors affecting efficacy and prognosis were assessed using univariate and multivariate Cox regression models. Results were considered statistically significant at *p* < 0.05. Analyses were performed with R version 4.3.1.

## 3 Results

### 3.1 Patient Demographics

A total of 252 patients were enrolled, and after the exclusion of 4 benign mesothelioma, 248 patients with MM were included in the study. Baseline characteristics were provided in Table 1. The median age was 60 (21–88) years old, 142 (42.7%) were males and 106 (57.3%) were females. Of 235 patients with ECOG PS score, 0–1 were recorded in 189 cases (80.4%) and ≥ 2 in 46 cases (19.6%). 100/228 (43.8%) patients had a history of smoking, 21/117 (17.9%) patients had an exacted asbestos exposure history, and 59/234 (25.2%) patients had a family history of tumors. Patients with stage I–II accounted for 21.3% (43/202), and III–IV for 78.7% (159/202). Pathological subtypes of epithelioid, biphasic, and sarcomatoid accounted for 89.2% (86/104), 4.8% (5/104), and 12.8% (13/104), respectively. The percentage of malignant pleural mesothelioma (MPM) was 89.2% (216/242) and 9.9% (24/242) for malignant peritoneal mesothelioma (MPeM).

**TABLE 1:** Baseline characteristics of the 248 malignant mesothelioma (MM) patients.

Characteristic	Total patients	*N* = 248 (percent)	Characteristic	Total patients	*N* = 248 (percent)
Gender	248		Clinical stage	202	
Female		106 (42.7%)	I–II		43 (21.3%)
Male		142 (57.3%)	III–IV		159 (78.7%)
Age	248	60 (21–88)	Calretinin	174	
< 65		152 (61.3%)	Positive		143 (82.2%)
65–74		70 (28.2%)	CK5/6	170	
75–84		25 (10.1%)	Positive		127 (74.7%)
≥ 85		1 (0.4%)	WT‐1	196	
Smoking history	228	100 (43.8%)	Positive		148 (75.5%)
Family history	234	59 (25.2%)	D2‐40	164	
ECOG PS	235		Positive		115 (70.1%)
≥ 2		46 (19.6%)	*BAP1* loss	17	
0–1		189 (80.4%)	Positive		5 (29.4%)
Asbestos exposure	117	21 (17.9%)	ALK	42	
Location	242		Positive		5 (11.9%)
Other		2 (0.8%)	PD‐L1 TPS	18	
Peritoneal		24 (9.9%)	< 1%		4 (22.2%)
Pleural		216 (89.2%)	≥ 1%		14 (77.8%)
Histological type	104				
Biphasic		5 (4.8%)			
Epithelioid		86 (82.7%)			
Sarcomatoid		13 (12.5%)			

Abbreviations: ALK: anaplastic lymphoma kinase; *BAP1*: BRCA1‐associated protein‐1; CK5/6: Cytokeratin 5/6; ECOG PS: Eastern Cooperative Oncology Group‐performance status; PD‐L1 TPS: programmed death‐ligand 1 tumor proportion score; WT‐1: Wilms' Tumor gene 1.

Compared with the non‐Asian MM patients in SEER database, our Chinese cohort had a lower median age of diagnosis (60 vs. 75 years), a higher proportion of youth (patients aged < 65, 61.3% vs. 20.9%) and female patients (42.7% vs. 25.0%), more advanced stage of III–IV (78.7% vs. 68.0%) (Table S1). There were similar percentage of the lesion location and histological subtype, with MPM accounting for 89.2% versus 83.3% and epithelioid subtype for 82.7% versus 83.9%, respectively.

As for the IHC biomarkers, the positive rate of Calretinin was 82.2% (143/174), CK5/6 was 74.7% (127/170), WT‐1 was 75.5% (148/196), and D2‐40 was 70.1% (115/164); 29.4% (5/17) of patients happened *BAP1* loss and 11.9% (5/42) patients had *ALK* rearrangements, which were all existed in epithelioid subtype. Positive expression of the PD‐L1 (≥ 1%) was 77.8% (14/18).

### 3.2 Clinical Outcomes

#### 3.2.1 Efficacy and Survival Analysis of Entire Cohort

**FIGURE 1:**
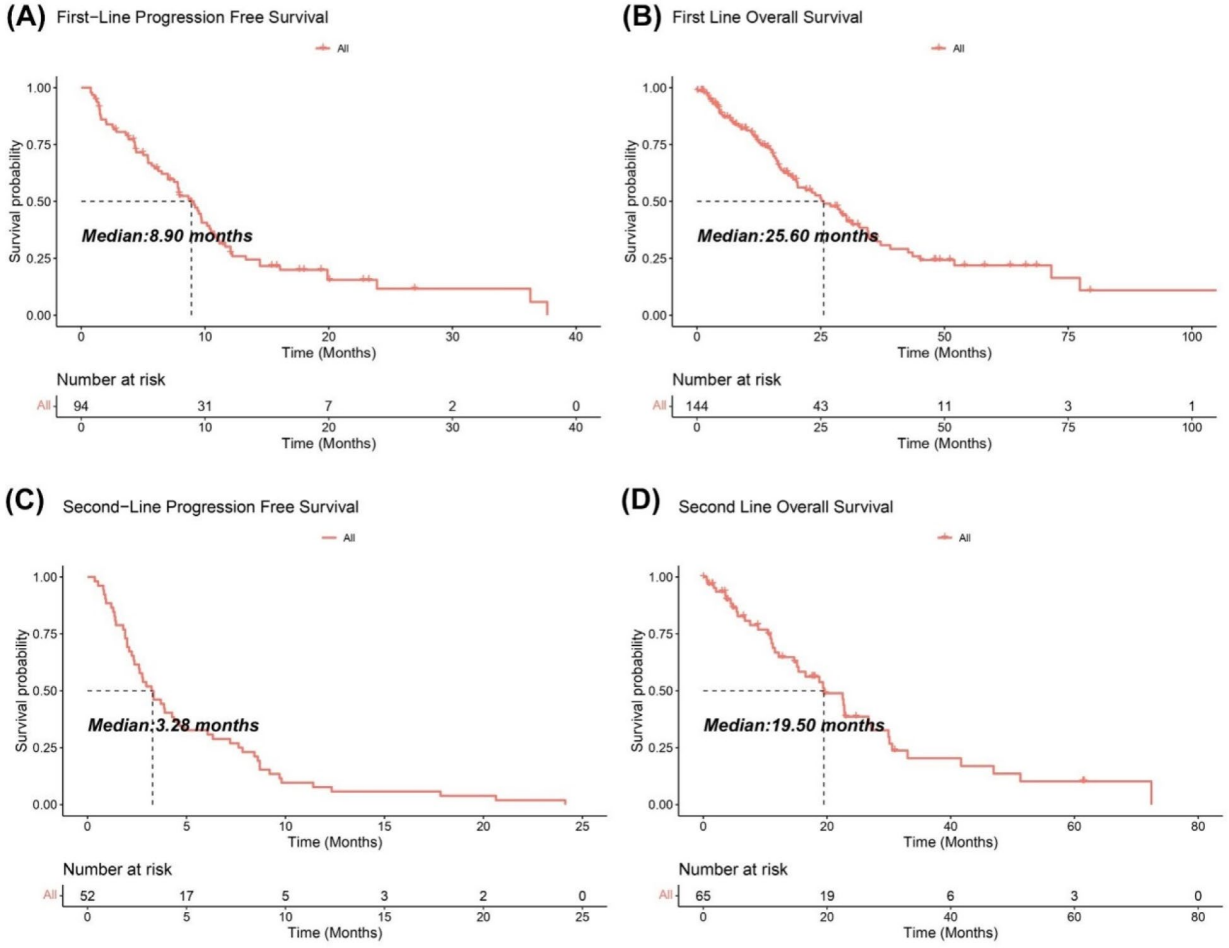
Survival analysis of Chinese population with malignant mesothelioma (MM). (A, B) Kaplan–Meier curves of the progression‐free survival (PFS) and overall survival (OS) for the first‐line treatment. (C, D) Kaplan–Meier curves of the PFS and OS for the second‐line treatment.

For the entire cohort, the first‐line treatment provided an ORR of 21.1% (28/133) and DCR of 94.7% (126/133), and acquired a mPFS of 8.90 months and a mOS of 25.60 months, respectively (Figure 1A,B). Patients received the second‐line treatment acquired an ORR of 7.7% (3/39) and DCR of 79.5% (31/39), and also a mPFS of 3.28 months and a mOS of 19.50 months (Figure 1C,D).

**FIGURE 2:**
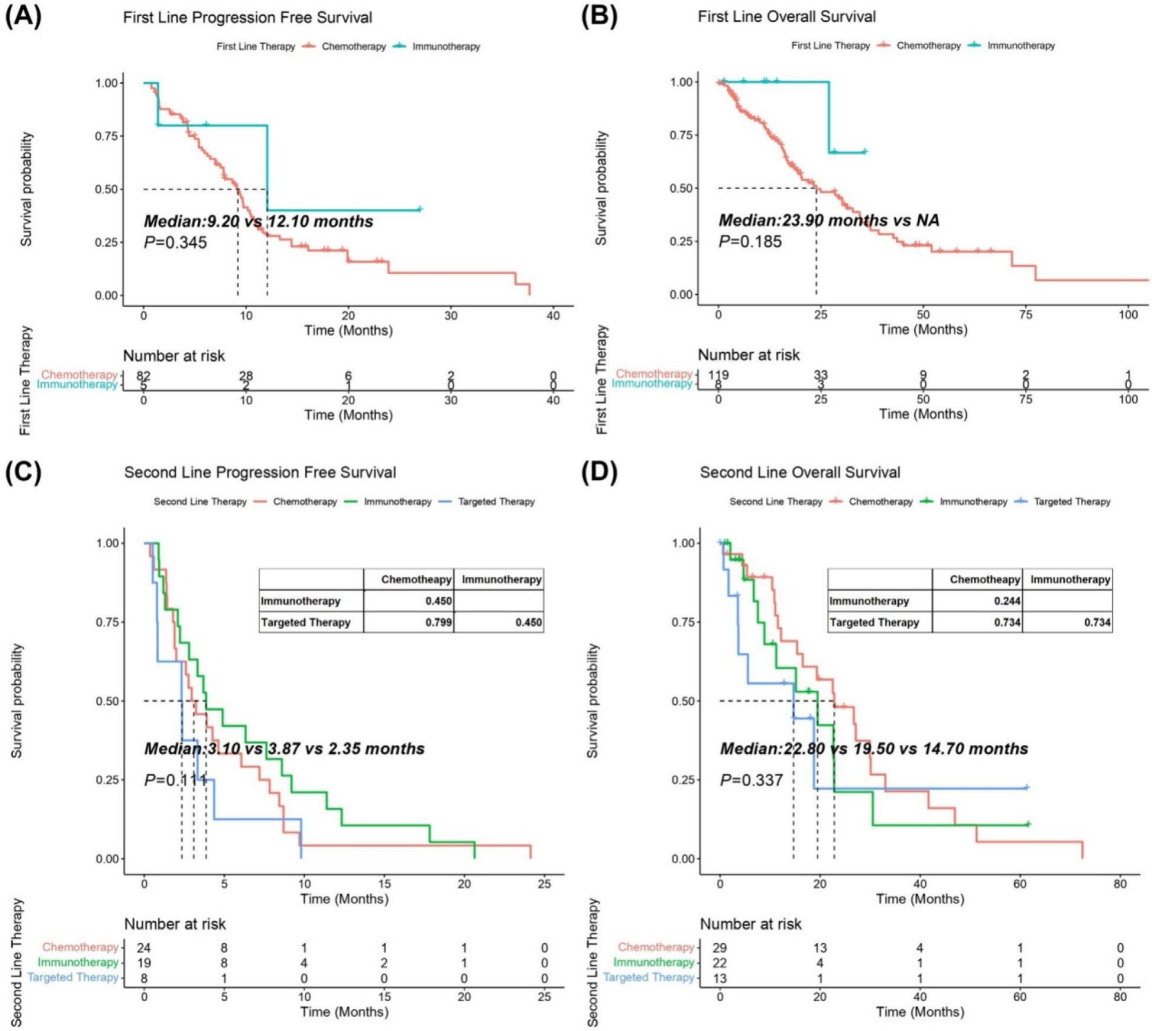
Comparison of survival time of MM patients received different regimens in the first‐line and the second‐line therapy. (A, B) Kaplan–Meier curves of PFS and OS for the first‐line treatment. (C, D) Kaplan–Meier curves of the PFS and OS for the second‐line treatment. PFS, progression free survival; OS, overall survival; Log‐rank test was used, *p* value < 0.05 was significant.

One hundred and eighty‐nine patients received first‐line treatment, of which 172 patients received chemotherapy, 13 for immunotherapy and 4 for targeted therapy. The ORR of immunotherapy group was relatively higher than that of chemotherapy group, although the difference was not significant, 33.3% (3/9) versus 20.5% (25/122) (*p* = 0.402). There was no difference of DCR between two groups, 100% (9/9) versus 95.1% (116/122), respectively. Immunotherapy showed a trend of prolonged mPFS (12.10 vs. 9.20 months, *p* = 0.345) and mOS (NA vs. 23.90 months, *p* = 0.185) compared with chemotherapy, although the differences were both not significant (Figure 2A,B).

A total of 87 patients received second‐line therapy, in which 37 received chemotherapy, 31 received immunotherapy and 19 received targeted therapy. The ORR of chemotherapy, immunotherapy and targeted therapy were 4.8% (1/21), 14.3% (2/14), and 0 (0/6), and the DCR were 81.0% (17/21), 78.6% (11/14), and 66.7% (4/6), respectively. There was no difference of mPFS between immunotherapy and chemotherapy (3.87 vs. 3.10 months, *p* = 0.450), which were both relatively prolonged compared with the targeted therapy (3.87 vs. 2.35 months, *p* = 0.450, 3.10 vs. 2.35 months, *p* = 0.799). Chemotherapy showed a trend to prolong the mOS compared with the immunotherapy (22.80 vs. 19.50 months, *p* = 0.244), while the targeted therapy provided the worst mOS of 14.7 months (Figure 2C,D).

#### 3.2.2 Hierarchical Analysis of the First Line Therapy in Chinese Population With MM

**FIGURE 3:**
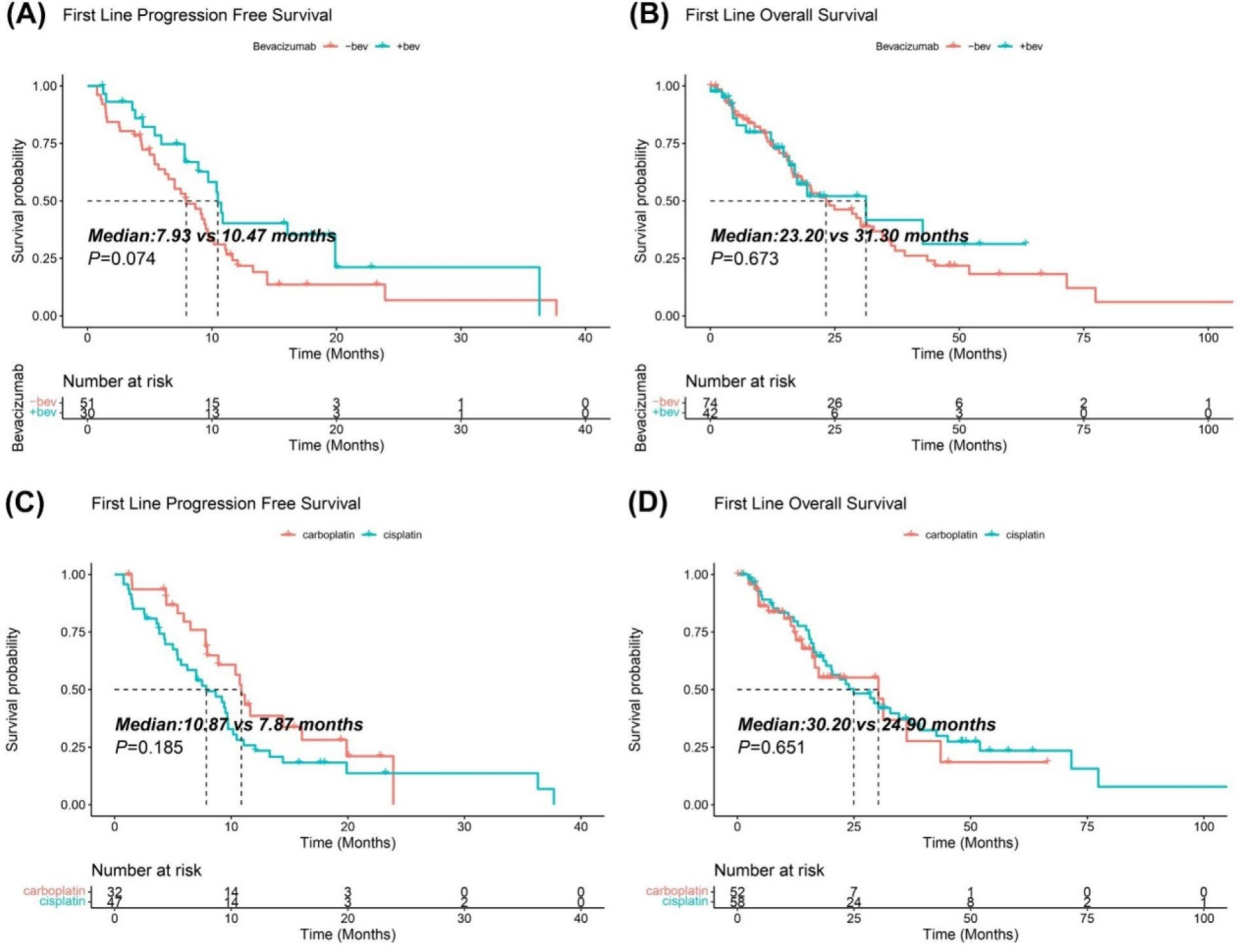
Hierarchical analysis of survival between different regimens in MM patients received the first‐line treatment. (A, B) Comparisons of PFS and OS between the chemotherapy combined with and without bevacizumab. (C, D) Comparisons of PFS and OS between carboplatin and cisplatin. PFS, progression free survival; OS, overall survival; Log‐rank test was used, *p* value < 0.05 was significant.

We evaluated the efficacy of bevacizumab combined with chemotherapy (PCB) and chemotherapy alone (PC) in patients received the first‐line treatment. There were no difference of ORR and DCR between PCB and PC groups, 20.5% (13/51) versus 28.9% (11/68) (*p* = 0.210, *χ* ^2^ = 1.571) and 96.1% (49/51) versus 94.1% (64/68) (*p* = 0.699), respectively. PCB group exhibited the potential to improve the mPFS (10.47 vs. 7.93 months, *p* = 0.074) and mOS (31.30 vs. 23.20 months, *p* = 0.673) than the PC group (Figure 3A,B).

We also compared the difference of efficacy between carboplatin and cisplatin in patients received the first‐line treatment. There were no differences of ORR and DCR between carboplatin and cisplatin groups, 22.4% (11/49) versus 19.7% (12/61) (*p* = 0.722, *χ* ^2^ = 0.127) and 93.9% (46/49) versus 98.4% (60/61) (*p* = 0.322), respectively. The carboplatin group had a relatively prolonged mPFS (10.87 vs. 7.87 months, *p* = 0.185) and mOS (30.20 vs. 24.90 months, *p* = 0.651) than the cisplatin group (Figure 3C,D).

**FIGURE 4:**
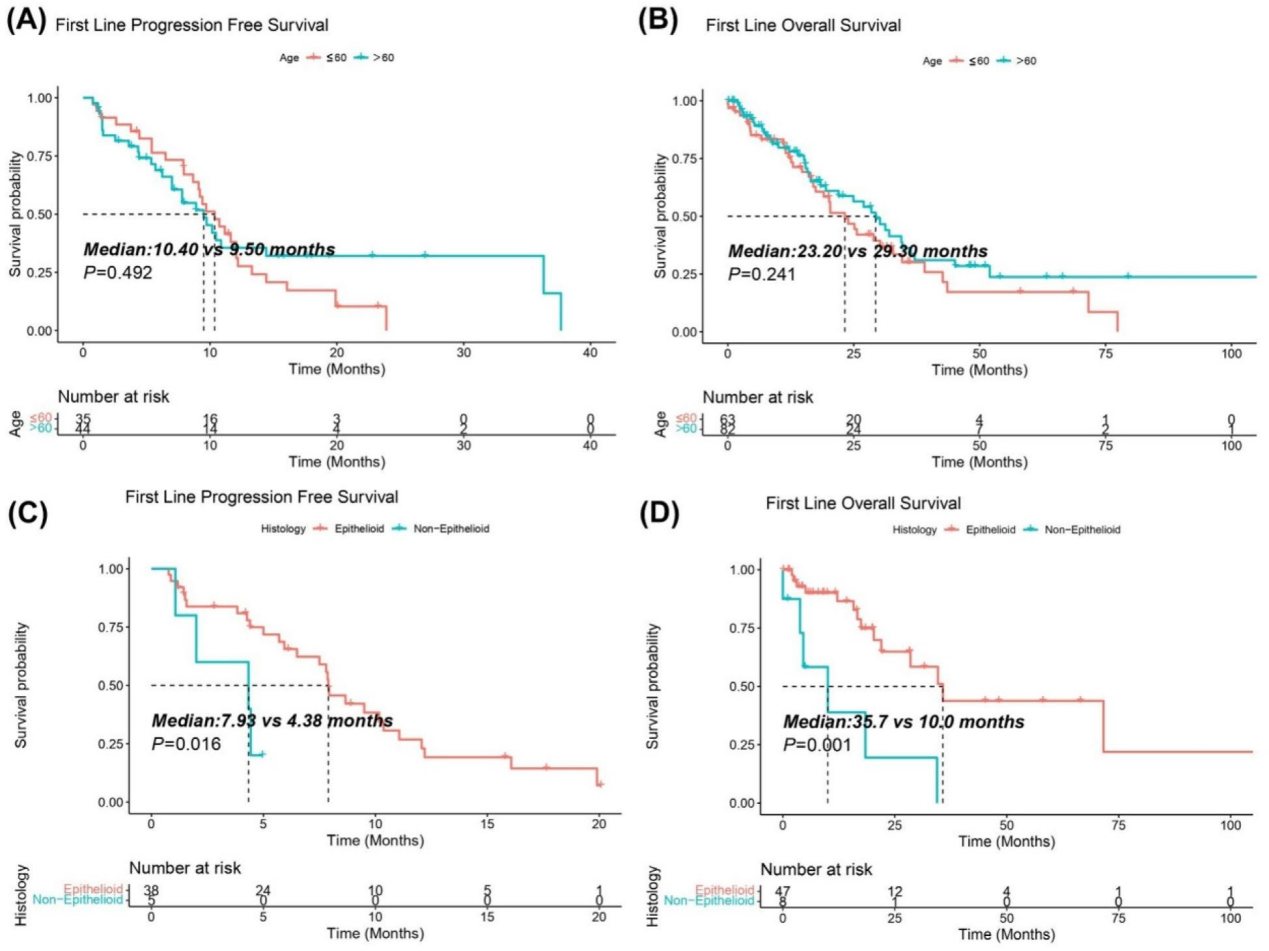
Hierarchical analysis of survival between different age groups and histology subtypes in MM patients received the first‐line treatment. (A, B) Comparisons of PFS and OS between the youth group (≤ 60 years) and the elderly group (> 60 years). (C, D) Comparisons of PFS and OS between epithelioid subtype and non‐epithelioid subtype. PFS, progression free survival; OS, overall survival; Log‐rank test was used, *p* value < 0.05 was significant.

We compared the survival time of the first‐line treatment in different age groups. There was no difference of mPFS between the youth group (≤ 60 years) and the elderly group (> 60 years), 10.40 versus 9.50 months, *p* = 0.492. While the youth group had an improved mOS compared with the elderly group, 29.30 versus 23.20 months, *p* = 0.241 (Figure 4A,B). Survival analysis was also conducted to compare the difference between histological subtypes. The epithelioid subtype provided significant survival benefits of mPFS and mOS than the non‐epithelioid, 7.93 versus 4.38 months(*p* = 0.016) and 35.7 versus 10.0 months (*p* < 0.001), respectively. (Figure 4C,D).

### 3.3 Cox Regression Analysis of Influence Factors Associated With the First‐Line PFS and OS

Multivariate Cox analysis showed that age and histological type were both the independent predictors for the first‐line PFS, and gender, histological type, and CK5/6 expression were all the independent prognosis factors of the first‐line OS (Table 2).

**TABLE 2:** Multivariate Cox regression associated with the first‐line PFS and OS in Chinese population with malignant mesothelioma (MM).

Variables	First‐line PFS		First‐line OS	
	HR (95%CI)	*p*	HR (95%CI)	*p*
Gender (male vs. female)	0.967 (0.916–1.021)	0.229	4.137 (1.107–15.458)	0.035
Age (≥ 60 vs. < 60)	4.952 (1.518–16.148)	0.008	0.996 (0.93–1.068)	0.917
ECOG PS (≥ 2 vs. 0–1)	4.314 (0.723–25.745)	0.109	—	—
Histological type (non‐epithelioid vs. epithelioid)	7.167 (1.296–39.634)	0.024	6.049 (1.538–23.787)	0.01
Calretinin (positive vs. negative)	—	—	0.656 (0.187–2.308)	0.512
CK5/6 (positive vs. negative)	—	—	0.108 (0.018–0.661)	0.016

*Note:* Multivariate Cox regression was used. *p* value < 0.05 was significant.

Abbreviations: CI, confidence interval; CK5/6, Cytokeratin 5/6; ECOG PS, Eastern Cooperative Oncology Group‐performance status; HR, hazard ratio; OS, overall survival; PFS, progression free survival.

### 3.4 *BAP1* Deletion and Its Effect on Survival in Chinese Patients With MM

**FIGURE 5:**
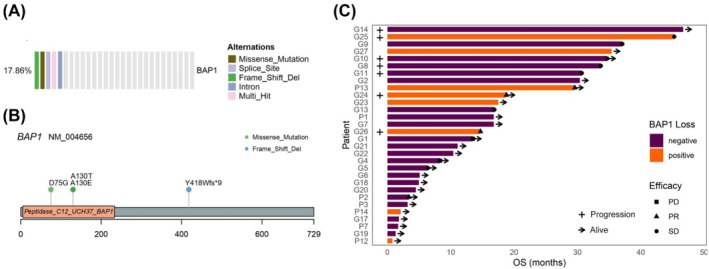
*BAP1* mutation and deletion status. (A) Heatmap summarizing the results the *BAP1* loss from the whole‐exome sequencing (WES) and the targeted panel sequencing. Each column represents a patient. (B) Location of somatic mutations in *BAP1* gene, the ubiquitin carboxy‐terminal hydrolase (UCH) domains were affected. (C) OS for different *BAP1* loss status. *BAP1*, breast cancer gene 1‐associated protein 1. PR: partial response; SD: stable disease; PD: progressive disease. The plus sign indicated disease progression, and the arrow indicated that the patient was alive at the last follow‐up.

The total mutation or deletion rate of *BAP1* was 17.78% (8/41), 3 patients were found to have *BAP1* mutation and 3 had BAP1 protein deletion and 2 had both *BAP1* mutation and deletion. The mutation rate of *BAP1* was 17.86% (5/28), among the 5 mutations detected, 3 of them affected the amino acid focusing on the fragment of Peptidase_C12_UCH37_BAP1, which referred to a domain of ubiquitin carboxy‐terminal hydrolase (UCH) (Figure 5A,B). The survival data of 30 patients have been collected and the bar plot showed that patients with *BAP1* loss were clustered at the side of longer OS.

## 4 Discussion

To our knowledge, this was the largest multi‐center real‐world study to analyze the clinicopathologic characteristics, efficacy, and survival of MM in Chinese population up to now. Compared with the former studies and non‐Asian population in SEER database, there were much lower ratio of asbestos exposure, similar incidence rate of pathological location and histological subtype, while more frequently happened in female, youth group and advanced stage in Chinese population. *BAP1* deletions ratio was much lower compared with that of the formerly reported, while the positive expression of ALK and PD‐L1 were more frequent. Immunotherapy showed the trendency to improve ORR, mPFS, and mOS compared with the chemotherapy group in the first‐line treatment. Hierarchical analysis showed that the epithelioid subtype and combination of bevacizumab and chemotherapy prolonged mPFS and mOS in the first‐line therapy. Age and histological subtype were independent predictors for the first‐line PFS, and histological subtype, gender, and CK5/6 were independently prognostic factors for first‐line OS.

Asbestos exposure is the most common cause of MM with a latency period of 30–50 years. In USA, the age‐specific incidence rates increase past age 60, from 0.5 to 1.24 cases (age 60–85 years) per 100 000, and reaches 6.34 cases per 100 000 in subjects over 85. The mean age of death from MM was 72.8 years with a male to female (M:F) mortality ratio of 4.2:1 [19]. In previous studies, more than 80% of MM could be attributed to asbestos exposure [15]. However, in the present study, only 17.9% of patients had a definite history of asbestos exposure, which might be related to the serious lack of awareness of asbestos exposure in Chinese patients, and also lead to the high rate of underdiagnosis and misdiagnosis of MM in China. The population distribution happened more frequently in female and youth group, and the mean age at diagnosis was younger compared with the United States. In addition, a large proportion of Chinese MM patients have a family history of tumor. This prevalence trend was in consistent with the incidence in Eastern China [5] and also the developing countries of South America [20]. Compared with the USA and Europe with high ratio of asbestos exposure, the incidence of MM in the developing countries might be correlated to genetic predisposition. There was a very high MM risk in family members with heterozygous for germline *BAP1* mutations [21]. Roushdy‐Hammady constructed genetic epidemiology maps to analyze a six‐generation extended pedigree of 526 individuals showed that mesothelioma was genetically transmitted, probably in an autosomal dominant way [22]. Further genetic evaluation is important for identifying the predisposed genomic profiling of MM in the Chinese population.

Currently, the diagnosis of MM still relies on histopathology, and the identification of MM‐specific IHC markers is important for the early diagnosis. The results of this Chinese cohort showed that the positive expression rate of Calretinin, CK5/6, WT‐1, and D2‐40 were much lower than those reported in previous studies. Calretinin is an important discriminative marker for MM whose high expression is associated with greater survival, and the positive rate of Calretinin in this study was lower than previous reports, 82.2% versus 96.7%, respectively [12]. CK5/6 is one of the sensitive IHC markers for MM, and the positive rate of MM in the Chinese population was 74.7%, which was much lower than the 89% ever reported [23]. The peptide derived from the WT‐1 protein is presented on the cell surface, which makes it an attractive target for immunotherapy. WT‐1 mimetic peptide vaccine has achieved good efficacy in phase II clinical trials [24], and the WT‐1 positive expression rate in the Chinese population in the present study was 75.5%, which was lower than the 87.7% in the existing study [12]. D2‐40 is a sensitive marker for epithelioid‐type MM, and its positive expression rate ranged from 80% to 100% [25], which was much higher than the D2‐40 positive rate in the present study of 70.1%. The rearrangement of the *ALK* gene is one of the common driver genes in lung adenocarcinoma, and the patients benefited significantly from the effects of the targeting of Alectinib and Crizotinib [26]. There had not been any relevant case reports of *ALK* gene rearrangement in Chinese MM patients. Five patients were found with *ALK* gene positive in this study, but only 1 of them was treated with CT‐707, a second‐generation *ALK* inhibitor. Genetic detection is rarely performed in MM patients due to the low availability of targeted therapies, while a genome evaluation and developing novel therapeutic targets could benefit more patients. The difference of clinicopathologic characteristics showed that Chinese MM patients had unique etiopathogenesis, which need further exploring of the genomics, pathologic features, and prognostic predictors of efficacy in the Chinese population.

In recent years, ICBs have shown good efficacy in MM treatment. In this study, the first‐line ICBs provided higher ORR, and longer mPFS and mOS than chemotherapy, which was consistent with the findings in Checkmate‐743 [9]. The ORR was significantly lower than the 40% for immunotherapy and 44% for chemotherapy in Checkmate‐743 study, which could be attributed to the fact that (1) the real‐world cohort was not as good as well‐designed clinical trials in terms of patient selection and quality control [27]; (2) there were more patients received mono‐ICBs or different categories of ICBs. However, the present study acquired more prolonged mPFS and mOS whether any regimens or overall population compared with Checkmate‐743 study, MERIT [28] and other studies [29]. It might be correlated with that population involved in the present study were much younger, more female, and non‐asbestos exposure. These survival benefits could also be observed in Latin American population, who were also in the developing countries [19, 20]. Of course, because only a small sample of patients were involved in immunotherapy, the above results needed to be further confirmed by the expanding sample size. According to Checkmate‐743, the non‐epithelioid type was more likely to benefit from immunotherapy. It was a pity that none of the 18 patients with non‐epithelioid subtypes in the study received immunotherapy. In addition, this study also demonstrated that most of patients in China received conventional first‐line pemetrexed combined with platinum, which was still a long way from achieving precise treatment. There was no difference of efficacy between chemotherapy and immunotherapy in second‐line treatment. The ORR and mPFS were both similar with the studies of PROMISE‐meso [30] and KEYNOTE‐158 [31], while the mOS was obviously prolonged compared with two studies. We speculated that it might be the similar causes like above.

Bevacizumab is a monoclonal antibody against VEGF‐A that inhibits angiogenesis and tumor growth, which been added to the first‐line option based on the result of MAPS [32] trials. In the present study, bevacizumab combined with chemotherapy (PCB) could significantly improve the mOS and mPFS compared with chemotherapy (PC) alone, which were consistent with MAPS trial. Treatment with pemetrexed plus cisplatin resulted in superior survival time, time to progression, and response rates compared with treatment with cisplatin alone in MM [33], while the difference between carboplatin and cisplatin in first‐line treatment was undefined. In the present study, carboplatin was superior to cisplatin in terms of mPFS, suggesting that carboplatin may be a suitable alternative to cisplatin‐based chemotherapy, though a phase 2 study [34] and a real‐world practice in United States [10] exhibited similar PFS between carboplatin and cisplatin.

Immunotherapy has been shown to prolong OS by about 4 months versus chemotherapy in MM [9], however, the predictive role of PD‐L1 for immunotherapy efficacy was controversial [35]; 77.8% patients of this study had a PD‐L1 positivity (PD‐L1 TPS ≥ 1%), much higher than the 18%–24% of a large international cohort [35]. However, immunotherapy was used in only 2 patients with positive expression of PD‐L1, one of whom had an expression of 60% and an efficacy assessment reached PR, while the other had an expression rate of 10% and the efficacy assessment was SD. It was suggested that there might be a correlation between PD‐L1 expression and efficacy, which needs to be further verified by enlarged sample size.

Studies have shown that some biomarkers were independent predictor of efficacy and prognosis factors of MPM, including *BAP1* expression, mesothelin, PD‐L1, but have not yet been applied in clinical practice [36, 37]. In this study, histological subtype was both an independent predictor and prognosis factor of first‐line PFS and OS, and gender was a prognostic factor for the first‐line OS, which were consistent with the previous study [38]. CK5/6 was a biomarker strongly associated with prognosis [39]. CK5/6 negativity in non‐muscle‐invasive papillary upper tract urothelial carcinoma hurt patient survival [40], but in invasive breast cancer, CK5/6 positivity harmed patient disease‐free survival (DFS) [41]. CK5/6 is one of the most sensitive immunohistochemical markers [23], however, there is a lack of evidence regarding the correlation between CK5/6 and survival in patients with MM. In the present study, we found for the first time that CK5/6 could be used as an independent prognosis marker of first‐line OS for Chinese MM.

*BAP1* is a nuclear deubiquitinating enzyme to form the BRCA1—BARD1—BAP1 complex, which is essential for homologous recombination (HR)‐mediated repair of DNA double‐strand breaks (DSBs) [42]. *BAP1* loss has been proven to be an independently favorable prognosis factor of OS in the first‐line chemotherapy of MM [37]. The total mutation and deletion rate of *BAP1* was 17.78% in the present study, which was much lower compared with the existing studies of 24%–60% [19, 43]. Although the limitation of case numbers, patients with *BAP1* expression deletions showed the trend for prolonged OS. The restricted sample size reflected the inadequate knowledge of genomics in Chinese population with MM, and clinical trials [44, 45] targeting *BAP1* have shown promising results suggesting the urgency of expanded sample size for further studies.

There were some shortcomings, first, only a few patients received immunotherapy, the relevant conclusions still needed to be further investigated by expanding the sample size; second, due to the limitation of retrospective study, there was a lack of records of adverse effects of the treatment, which leads to an inadequate discussion on the safety of the treatment; third, only a small part of patients underwent NGS and IHC evaluation of genetic alteration and expression of ALK and PD‐L1. It is necessary to conduct prospective multi‐center studies to further clarify the etiology and clinical treatment traits of MM in the Chinese population.

In conclusion, this study was the largest Chinese cohort with MM to detail the clinicopathologic characteristics and survival information up to now. Chinese population with MM exhibited unique clinicopathologic characteristics and relatively prolonged survival time. The first‐line immunotherapy showed the trendency to improve ORR, and to prolong mPFS and mOS. The combination of bevacizumab with chemotherapy improved the first‐line mOS and mPFS. Age and histological type were predictors for PFS, and gender, histological type, and CK5/6 were independent prognostic factors for survival. *BAP1* mutations or deletions present were correlated with the prolonged OS.

## Author Contributions

Conception and design: Chenrui Sun, Xue Yang, Jia Zhong, and Jiangyong Yu. Acquisition of data: Xue Yang, Jia Zhong, Runting Kang, Zhixin Bie, Bin Ai, Juanjuan Liu, and Zitong Zheng. Analysis and interpretation of data: Chenrui Sun, Zitong Zheng, and Haolan Liu. Writing, review, and/or revision of the manuscript: Chenrui Sun, Xue Yang, Jiangyong Yu, and JiaZhong. Study supervision: Jiangyong Yu and Jia Zhong. All authors read and approved the final manuscript.

## Ethics Statement

This retrospective study was approved by the medical ethics committee of Beijing Hospital (2022BJYYEC‐211‐02).

## Conflicts of Interest

The authors declare no conflicts of interest.

## Supporting information


**Table S1.** Comparison of characteristics of patients between non‐Asian from Surveillance, Epidemiology, and End Results (SEER) and Chinese cohort.

## Data Availability

The data that support the findings of this study are available from the corresponding author upon reasonable request.
