# Supplementary material for: A Multi‐Center Real‐World Study of Clinicopathologic Characteristics and Efficacy of the Malignant Mesothelioma in Chinese Population
Source: Thorac Cancer. 2025 Feb 12;16(3):e15533. doi: 10.1111/1759-7714.15533 (PMC11821455; doi:10.1111/1759-7714.15533)
Supplement: Supplementary file 1 — Table S1. Comparison of characteristics of patients between non‐Asian from Surveillance, Epidemiology, and End Results (SEER) and Chinese cohort. [file TCA-16-e15533-s001.docx]

Supplementary table 1 Comparison of characteristics of patients between non-Asian from Surveillance, Epidemiology, and End Results (SEER) and Chinese cohort

| **Characteristic** | | **Non-Asian from SEER**^1^ | **Chinese cohort** |
| --- | --- | --- | --- |
| Age | | n=14,328 | n=248 |
| median age | | 75 (66, 82) | 60 (55, 68) |
| <65 | | 2,997 (20.9%) | 152(61.3%) |
| 65-74 | | 3,963 (27.7%) | 70(28.2%) |
| 75-84 | | 5,154 (36.0%) | 25(10.1%) |
| ≥85 | | 2,214 (15.5%) | 1(0.4%) |
| Gender | | n=14,328 | n=248 |
| Female | | 3,589 (25.0%) | 106 (42.7%) |
| Male | | 10,739 (75.0%) | 142 (57.3%) |
| Disease Location | | n=12,515 | n=242 |
| Other | | 1,086 (8.7%) | 2 (0.8%) |
| Peritoneum | | 1,003 (8.0%) | 24 (9.9%) |
| Pleura | | 10,426 (83.3%) | 216 (89.2%) |
| Histology | | n=6,378 | n=104 |
| Biphasic | | 1,026 (16.1%) | 5 (4.8%) |
| Epithelioid | | 5,352 (83.9%) | 86 (82.7%) |
| Sarcomatoid | | 0 | 13 (12.5%) |
|  |  |  |  |
